# Diagnostic Laparoscopy with Ultrasound Still Has a Role in the Staging of Pancreatic Cancer: A Systematic Review of the Literature

**DOI:** 10.1155/2016/8092109

**Published:** 2016-03-30

**Authors:** Jordan Levy, Mehdi Tahiri, Tsafrir Vanounou, Geva Maimon, Simon Bergman

**Affiliations:** ^1^Division of General Surgery, Jewish General Hospital, McGill University, Montreal, QC, Canada H3T 1E2; ^2^Lady Davis Institute for Medical Research, Montreal, QC, Canada H3T 1E2

## Abstract

*Background*. The reported incidence of noncurative laparotomies for pancreatic cancer using standard imaging (SI) techniques for staging remains high. The objectives of this study are to determine the diagnostic accuracy of diagnostic laparoscopy with ultrasound (DLUS) in assessing resectability of pancreatic tumors.* Study Design*. We systematically searched the literature for prospective studies investigating the accuracy of DLUS in determining resectability of pancreatic tumors.* Results*. 104 studies were initially identified and 19 prospective studies (1,573 patients) were included. DLUS correctly predicted resectability in 79% compared to 55% for SI. DLUS prevented noncurative laparotomies in 33%. Of those, the most frequent DLUS findings precluding resection were liver metastases, vascular involvement, and peritoneal metastases. DLUS had a morbidity rate of 0.8% with no mortalities. DLUS remained superior to SI when analyzing studies published only in the last five years (100% versus 81%), enrolling patients after the year 2000 (74% versus 58%), or comparing DLUS to modern multidimensional CT (100% versus 78%).* Conclusion*. DLUS seems to still have a role in the preoperative staging of pancreatic cancer. With its ability to detect liver metastases, vascular involvement, and peritoneal metastases, the use of DLUS leads to less noncurative laparotomies.

## 1. Introduction

Pancreatic cancer represents an aggressive disease that is resectable in only 10–20% of patients at the time of diagnosis [1, 2]. While resection can be curative in some, it may also be abandoned intraoperatively due to the presence of occult advanced disease [3]. Careful selection of patients for surgery is important in order to avoid unnecessary procedures and their associated morbidities. In addition, with the advent of minimally invasive procedures for symptomatic relief and palliation, such as endoscopic and percutaneous biliary stenting and laparoscopic duodenal and biliary bypass, the need to correctly identify unresectable patients prior to laparotomy has been further emphasized [4].

Diagnostic laparoscopy (DL) was introduced in many preoperative staging algorithms for pancreatic carcinoma over 20 years ago [5]. Its value seemed to have been considerably enhanced with the adjunct of laparoscopic ultrasound (LUS) [4, 6]. Despite the growing body of research in the use of diagnostic laparoscopy with ultrasound (DLUS) for preoperative staging of pancreatic cancers, its application remains controversial [7]. Several studies support its use, as it is a sensitive tool in detecting small hepatic lesions, vascular invasion, and malignant lymphadenopathy [8]. However, many have argued, especially with the advent of multidimensional computed tomography (CT), that standard imaging (SI) modalities may be sufficient and just as reliable in staging of pancreatic cancer, obviating the need of an additional operative procedure [9].

We performed a systematic review of prospective studies investigating the use of DLUS in staging pancreatic cancer. The objectives of this study are (1) to determine the diagnostic accuracy of DLUS in assessing resectability of pancreatic tumors, (2) to compare the reported resection rates of DLUS to standard preoperative imaging, and (3) to determine how the accuracy of these modalities has evolved over time.

## 2. Methods

### 2.1. Data Sources and Searches

A focused literature search using Medline and EMBASE databases, through June 2014, was conducted. Prospective studies evaluating the accuracy of diagnostic laparoscopy followed by laparoscopic ultrasound in determining resectability of pancreatic cancer were included. The search strategy combined the terms “laparoscopic ultraso^*∗*^” and “pancrea^*∗*^” and “cancer” or “tumor^*∗*^” or “malignancy” and “stage” or “staging” in the English language. This strategy was complemented by manually searching the references of the studies identified in the primary search. Study eligibility criteria were (1) that it was prospective; (2) that its objective was to investigate the accuracy of DLUS in determining resectability of pancreatic tumors; (3) that it reported intraoperative DLUS findings; and (4) that surgery was considered the gold standard for resectability.

### 2.2. Data Extraction

Data from each study was independently extracted by two reviewers. Disagreements were resolved by consensus or, when necessary, by a third reviewer. The reviewers systematically extracted information on author, date of publication, institution, study design, enrolment years, patient demographics, type of preoperative imaging, laparoscopic ultrasound probe and monitor specifications, morbidity associated with DLUS, and failure rates in performing DLUS. The reviewers also extracted statistical data, including sensitivity, specificity, and predictive values of DLUS and SI. We respected the following rigorous criteria for our analysis: (1) all patients declining or unfit (determined by the surgical team at that time) for DLUS or laparotomy were excluded. (2) All patients in whom laparoscopic ultrasound was not achieved were excluded, unless diagnostic laparoscopy had already proven unresectability before LUS was required. (3) In certain studies, patients were classified as “doubtfully resectable”; those patients were treated similarly to the resectable group and were thus included in our study as such. (4) Benign lesions discovered at DLUS or laparotomy were considered as “resected” for the purpose of the analysis.

### 2.3. Statistical Analysis

Both imaging techniques, DLUS and SI, are being used to determine the resectability of a pancreatic cancer. Hence, for our purposes, the term “true positive” refers to a cancer that was deemed resectable by a staging technique and was actually resected. Similarly, a “true negative” refers to a cancer deemed unresectable by SI or DLUS and confirmed as unresectable according to operative findings, cytopathology, frozen section, or grossly suspicious findings during either staging technique. Sensitivity is defined as the number of true positives over the number of resectable cancers. Specificity is defined as the number of true negatives over the total number of unresectable cancers. The positive predictive value is the number of true positives over the total number of cancers deemed resectable by imaging. Negative predictive value is the number of true negatives over the total number of cancers deemed unresectable by imaging. Our measure of resection rate is equivalent to the positive predictive value, as defined above. To calculate the overall resection rate across all applicable studies, the data were weighted according to each study's sample size.

## 3. Results

### 3.1. Study Selection and Baseline Characteristics

**Figure 1:**
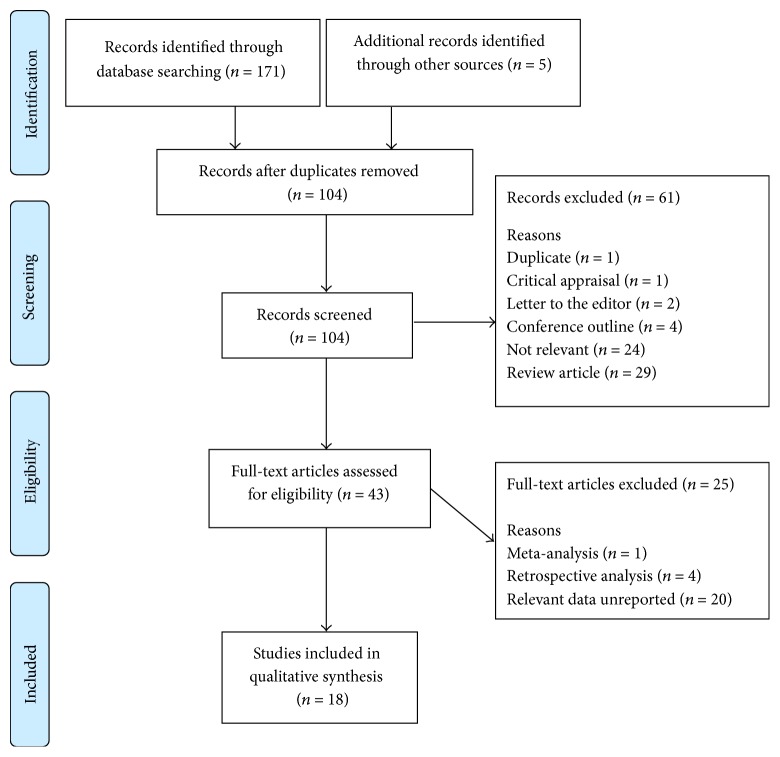
Search diagram.

Study selection occurred according to the Preferred Reporting Items for Systematic Review and Meta-Analyses (PRISMA) diagram (Figure 1). The search initially identified a total of 99 abstracts, with additional five abstracts found after a manual search through the references. These abstracts were reviewed and screened for relevance. 43 full-text and potentially relevant articles were retrieved and evaluated for eligibility following exclusion of review articles (*n* = 29), nonrelevant articles (*n* = 24), conference outlines or abstracts (*n* = 4), letters to the editor (*n* = 2), critical appraisal (*n* = 1), and duplicate abstract (*n* = 1). Of the 43 full-text studies retrieved, 18 studies met the inclusion criteria and were included in the analysis. Studies were excluded because they did not provide relevant analytical data necessary for the calculation of the sensitivity and specificity of DLUS as a diagnostic tool (*n* = 20) or were not prospective studies (*n* = 5). One of the 18 prospective studies included in the systematic review reported a two-part study occurring at different times on different study populations [10]. It was thus considered as two separate studies, bringing the total to 19 prospective studies and 1,573 patients.

Eleven of 19 studies were published after January 1, 2000. The average patient age ranged from 55 to 66 years old. The percentage of male patients ranged from 25 to 64%. The location of the pancreatic tumor was found most commonly in the pancreatic head, followed by the ampullary region, body, and tail, and rarely in the uncinate process (Table 1).

### 3.2. Execution of Preoperative Staging

CT scan was the investigation of choice in the assessment of resectability in all but one study (18/19), which was completed in a center where mesenteric angiography was frequently performed [25]. 79% (15/19) of studies reported using at least one additional staging procedure following CT [4, 6, 11, 13–20, 22–25]: abdominal ultrasound (15/19) [4, 6, 11, 13–20, 22–25], endoscopic retrograde pancreatography (10/19) [4, 6, 14, 16–20, 22, 24], endoscopic ultrasound (5/19) [13, 14, 18, 19, 23], visceral angiography (7/19) [4, 6, 14, 16, 23–25], and magnetic resonance imaging (MRI) (5/19) [14–17, 20], although the additional procedures were not performed in all patients.

Diagnostic laparoscopy was first carried out to explore the peritoneal cavity in search of malignant ascites, peritoneal metastases, visceral implants, or suspicious lymph nodes. The LUS probe was then inserted. Most often, the probe used linear array with a frequency of 5–7.5 MHz and frequently had Doppler capabilities. The liver was scanned in search of undiagnosed micrometastases and the biliary tree explored for any abnormalities. The pancreas was scanned to better characterize the primary lesion and determine local extensions into peripancreatic tissues including duodenum, mesocolon, stomach, and spleen. In less than one-third of the studies did the authors explicitly report exploring the lesser sac by retroduodenal or infragastric approaches. Blood vessels, including the celiac axis, superior mesenteric artery, and the portal venous system, were characterized according to their relation to the tumor and whether they were encased, thrombosed, stenosed, infiltrated, or frankly invaded. Associated lymph node basins were also investigated.

Nine studies described DLUS timing [6, 11–13, 16, 18, 19, 21, 24]. In five studies it occurred as a separate procedure prior to laparotomy [6, 12, 18, 19, 24]; in two studies it occurred in the same setting immediately prior to laparotomy [11, 16]. In two studies it occurred both immediately before and as a separate procedure [13, 21]. Procedure time varied between 15 and 90 minutes depending on surgeon experience and whether biopsies and lesser sac dissection were performed.

### 3.3. Morbidity and Mortality

Complication rates were minimal at 0.8% (9/1076), including 2 port-site hemorrhages, 2 episodes of pancreatitis, 2 wound infections, 1 enterotomy, 1 aspiration pneumonia, and 1 bile leak following biopsy [4, 6, 16, 17, 24]. There were no procedure-related mortalities.

### 3.4. Resectability Criteria

Nonresectability criteria differed between studies. All studies considered liver and peritoneal and other distant metastases unresectable. Seven studies only considered distant lymphadenopathy as unresectable [11, 13, 14, 16, 17, 21, 22] while two studies included regional involvement [4, 19]; the rest of the studies did not specify. Size was only considered in three studies [4, 12, 25]. Most studies considered any vascular involvement as unresectable, except four studies in which some degree of portal vein or superior mesenteric vein was considered resectable [14, 16, 22, 24]. All but one study [11] discussed confirmation of nonresectability due to liver, peritoneal, or lymph node metastases by biopsy proven histopathology.

### 3.5. Rates of Resection

Studies including data on SI are summarized in Table 2. CT was used in 99.7% (1569/1573) of patients to determine resectability; 4 patients underwent angiography without CT. Of these, the data for 1442 patients from 15 studies were available for analysis [4, 6, 10–14, 16, 18, 19, 21, 22, 24, 25]. Eight of 15 studies only included “SI resectable” patients in their analysis without presenting the initial study population screened by SI, precluding a sensitivity and specificity analysis [4, 10, 11, 18, 21, 24, 25]. Following imaging, 911 patients were considered resectable and of these, only 505 were resected at laparotomy, corresponding to a resection rate of 55% (29%–85%) [4, 6, 10–14, 16, 18, 19, 21, 22, 24, 25].

Table 3 summarizes DLUS data. 1076 patients were initially considered for DLUS. However, five patients declined further investigations and were excluded from the study; failures due to dense adhesions occurred in nine patients, while 12 patients were deemed unfit for surgery and were also excluded from formal analysis. Ultimately, 1050 patients were investigated using DLUS. 646 patients were deemed resectable and 513 were finally resected, corresponding to a resection rate of 79% (41%–100%). Of note, even those studies employing additional diagnostic procedures following CT did not show superior accuracy than DLUS. Such complementary studies, such as EUS, once represented an important role in pancreatic cancer staging and have now fallen out of favor with certain institutions recommending against its routine use in staging [26].

### 3.6. DLUS versus SI

14 studies presented data on SI and DLUS findings in a sequential manner such that the study population could be followed up from SI to DLUS [4, 6, 10–14, 16, 18, 19, 21, 24, 25]. In 781 patients deemed resectable by SI, DLUS correctly prevented noncurative laparotomies in 254 (33%). In this group, the most common findings precluding resection were liver metastases, vascular involvement, and peritoneal metastases.

### 3.7. DLUS versus DL

The added benefit of laparoscopic ultrasound (LUS) to diagnostic laparoscopy (DL) was investigated and clearly reported in three studies. In these studies, diagnostic laparoscopy with ultrasound (DLUS) identified 64 unresectable patients, of which 37 were discovered using ultrasound after being overlooked by diagnostic laparoscopy (DL) alone. Signifying that 58% of these accurate staging procedures were directly attributable to the addition of ultrasound to diagnostic laparoscopy. The findings precluding resection in these 37 patients were 17 vascular involvements, 14 liver metastases, 5 malignant lymphadenopathies, and 1 transverse mesocolon invasion [6, 13, 21].

### 3.8. Controlling for Advances in Diagnostic Imaging

As imaging studies have improved substantially in recent years, subgroup analyses of studies published in the last five years, enrolling patients after 2000 and those using multidimensional CT (MDCT), were carried out. In studies published between 2009 and 2014 (two studies), the resection rates using DLUS and SI were 100% and 81% (78%–85%), respectively [10, 11]. In those studies enrolling patients only after the year 2000 (four studies), the resection rates were 74% (54%–100%) and 58% (29%–85%) for DLUS and SI, respectively [10–13]. In the only prospective study specifically comparing DLUS to multidimensional CT (and no previous model of CT), the resection rates were 100% and 78%, respectively [10].

## 4. Discussion

Currently, DLUS is not routinely used in preoperative staging of pancreatic tumors. Some institutions selectively incorporate it into staging protocols, while others do not use it at all. Our study was designed to determine the accuracy of DLUS in determining resectability of pancreatic tumors. We included only the most rigorous prospective studies, in which DLUS, SI, and laparotomy findings were clearly reported.

Overall, by weighted analysis, DLUS improved the resection rate of pancreatic malignancies from 55% to 79% with no increase in mortality and a 0.8% complication rate. DLUS remained more accurate when restricting our analysis to more recent studies, in which SI had presumably improved.

A meta-analysis published in 2010 evaluating the role of DL and LUS in the preoperative staging of pancreaticobiliary cancer demonstrated that it improved resection rates of pancreatic malignancies from 61% to 80% [27]. These results are largely consistent with our systematic review. Our study differs in that we included only prospective studies and focused on comparing operative findings and resection rates following DLUS to SI. In addition, we have updated the literature search with all eligible studies published after the meta-analysis.

### 4.1. Modernized Standard Imagine

It is possible that the studies included in this systematic review are not representative of modern staging techniques, as they did not all employ MDCT. It is important to acknowledge that modern techniques for CT imaging offer higher-resolution images with more detail of vascular involvement and metastatic disease. Advances in CT imaging, namely, multiphase imaging technique including noncontrast, arterial, pancreatic parenchymal, and portal venous phases with cuts less than 3 mm through the abdomen, have improved its ability to predict resectability of pancreatic tumors [28, 29]. A prospective study comparing MDCT Angiography with MDCT 3D Reconstruction reported resection rates of 94% and 100%, respectively. However, MDCT Angiography also overestimated unresectability in 32% of patients, which may be in part due to overestimating vascular invasions [30]. The authors suggest that older grading schemes like those presented by Lu et al. [31] and Loyer et al. [32], which assess circumferential contiguity, tissue planes, mass effects, and occlusions, may be improved by visualizing tumor infiltration and vascular smoothness. An assessment readily made by LUS.

A study investigating MDCT for pancreatic head tumors found that only 40% of their “CT resectable” group was resected and that this was due to MDCT underestimating vascular involvement and local invasion. A subgroup analysis of patients that were unequivocally resectable improved the resection rate to 56% [33].

The use of MRI has increased dramatically in recent years and is considered by some to be standard of care along with MDCT cross-sectional imaging [34]. Using MRI with a pancreas protocol, at a high volume center, leads to a resection rate of 73%. The most common causes of intraoperative unresectability were vascular involvement and distant metastases, two findings aptly diagnosed by DLUS [29].

### 4.2. Timing and Cost Analysis

We believe that the optimal approach to include DLUS in the staging protocol is immediately prior to planned resection, which would minimize risks related to a second surgical procedure and general anesthesia. It may prove to be cost-effective as the patient would ultimately spend fewer days in hospital and most importantly decrease theoretical risk of progression in between procedures and delay in chemoradiation [35, 36]. In a recent cost-efficacy analysis of diagnostic laparoscopy prior to laparotomy for pancreatic cancer, the authors found that the total cost for introducing diagnostic laparoscopy was 1,480$ less per patient and provided better quality of life [37].

## 5. Limitations

This study has several limitations. The studies were heterogeneous, in their resectability criteria, use of multimodal imaging protocols, and the quality of their CT technology. In recent years there has been a paucity of literature on the subject and thus direct comparison of DLUS with more modern SI techniques is not possible. An important issue with DLUS is that the excellent results reported here may not be easily transferable to other centers where experience with this technique may be limited. The true benefit of DLUS may be difficult to achieve in all cases given the required expertise to perform and interpret this test correctly. In one study spanning three years, the average time to perform DLUS with lesser sac dissection in 67 patients was 30 minutes. The time to perform improved to 21 from 39 minutes in the last six months of the study [19].

## 6. Conclusion

Based on the highest quality studies available at this time, DLUS seems to still have a role in the preoperative staging of pancreatic cancer alongside SI techniques. With its ability to detect occult liver metastases, vascular involvement, and peritoneal metastases, the use of DLUS may lead to less noncurative laparotomies. Further research is warranted to compare DLUS to Pancreas Protocol MDCT and MRI.

## Figures and Tables

**Table 1:** Study characteristics.

Author	Year of publication	Years of enrolment	Study design	Country	Sample size	Mean age	% male	Location/tumor type
Barabino et al. [10]	2011	1995–1999	Prospective	Italy	40	NR	NR	Periampullary 40
Barabino et al. [10]	2011	2002–2007	Prospective	Italy	64	NR	NR	Periampullary 64
Piccolboni et al. [11]	2010	2005–2008	Prospective	Italy	48	NR	NR	NR
Doucas et al. [12]	2007	2001–2004	Prospective	UK	100	63	52%	Head 90, body, or tail 10
Fristrup et al. [13]	2006	2002–2004	Prospective	Denmark	148	66^*∗*^	54%	NR
Doran et al. [14]	2004	1997–2002	Prospective	UK	239	64^*∗*^	60%	NR
Zhao et al. [15]	2003	NR	Prospective	China	22	55	64%	Head 22
Kwon et al. [16]	2002	1996–2000	Prospective	Japan	118	59	64%	Head 39, body 13
Lavonius et al. [17]	2001	1997–1999	Prospective	Finland	27	63	48%	Head 21, body 2, chronic pancreatitis 4
Taylor et al. [18]	2001	1996–2000	Prospective	UK	51	66	57%	Head 42, ampullary 9
Schachter et al. [19]	2000	1996–1999	Prospective	Israel	94	63	46%	Head 40, body, or tail 19 UP 5, ampullary 3
Velasco et al. [20]	2000	NR	Prospective	USA	33	NR	NR	NR
Norton et al. [21]	1999	NR	Prospective	USA	50	NR	NR	NR
Minnard et al. [22]	1998	1993–1995	Prospective	USA	90	65^*∗*^	47%	Head 64, body 19, ampullary 4, tail 3
Champault et al. [23]	1997	1994–1996	Prospective	France	26	61	46%	Head 26
Pietrabissa et al. [24]	1996	1994-1995	Prospective	Italy	21	65	62%	Head 14, body, or tail 7
Bemelman et al. [6]	1995	1993-1994	Prospective	Netherlands	350	NR	NR	Head 60, ampullary 13
John et al. [4]	1995	1991–1993	Prospective	UK	40	59^*∗*^	45%	NR
Murugiah et al. [25]	1993	1991-1992	Prospective	UK	12	58	25%	Head 12

^*∗*^Median age.

AdenoCA = adenocarcinoma, NOS = not otherwise specified, CCA = cholangiocarcinoma, NET = neuroendocrine tumor, UP = uncinate process, NR = not reported.

**Table 2:** Analysis of SI.

Author	Year	# receiving SI	Analysis sample	Resectability			
				Sensitivity	Specificity	PPV	NPV
Barabino et al. [10]	2011	40^*∗*^	40	NA	NA	33% (13/40)	NA
Barabino et al. [10]	2011	64^*∗*^	64	NA	NA	78% (50/64)	NA
Piccolboni et al. [11]	2010	48^*∗*^	48	NA	NA	85% (41/48)	NA
Doucas et al. [12]	2007	100	94	71% (20/28)	26% (17/66)	29% (20/69)	68% (17/25)
Fristrup et al. [13]	2006	148	148	100% (38/38)	64% (70/110)	49% (38/78)	100% (70/70)
Doran et al. [14]	2004	239	227	96% (127/132)	46% (44/95)	71% (127/178)	90% (44/49)
Zhao et al. [15]	2003	22	NR	NA	NA	NA	NA
Kwon et al. [16]	2002	118	118	100% (39/39)	84% (66/79)	75% (39/52)	100% (66/66)
Lavonius et al. [17]	2001	27	NR	NA	NA	NA	NA
Taylor et al. [18]	2001	51^*∗*^	49	NA	NA	53% (26/49)	NA
Schachter et al. [19]	2000	94	94	100% (33/33)	44% (27/61)	49% (33/67)	100% (27/27)
Velasco et al. [20]	2000	33	NR	NA	NA	NA	NA
Norton et al. [21]	1999	50^*∗*^	50	NA	NA	52% (26/50)	NA
Minnard et al. [22]	1998	90	90	100% (40/40)	34% (17/50)	55% (40/73)	100% (17/17)
Champault et al. [23]	1997	26	NR	NA	NA	NA	NA
Pietrabissa et al. [24]	1996	21^*∗*^	21	NA	NA	62% (13/21)	NA
Bemelman et al. [6]	1995	350	347	100% (22/22)	85% (277/325)	31% (22/70)	100% (277/277)
John et al. [4]	1995	40^*∗*^	40	NA	NA	30% (12/40)	NA
Murugiah et al. [25]	1993	12^*∗*^	12	NA	NA	42% (5/12)	NA

^*∗*^Size of initial population screened not available. Only patients deemed resectable as per SI were included.

NA = Not applicable, NR = Not Reported.

**Table 3:** Analysis of DLUS.

Author	Year	Analysis sample	Resectability			
			Sensitivity	Specificity	PPV (resection rate)	NPV
Barabino et al. [10]	2011	40	100% (13/13)	93% (25/27)	87% (13/15)	100% (25/25)
Barabino et al. [10]	2011	9	100% (1/1)	100% (8/8)	100% (1/1)	100% (8/8)
Piccolboni et al. [11]	2010	48	100% (41/41)	100% (7/7)	100% (41/41)	100% (7/7)
Doucas et al. [12]	2007	94	100% (28/28)	64% (42/66)	54% (28/52)	100% (42/42)
Fristrup et al. [13]	2006	78	100% (38/38)	65% (26/40)	73% (38/52)	100% (26/26)
Doran et al. [14]	2004	227	98% (130/132)	57% (54/95)	76% (130/171)	96% (54/56)
Zhao et al. [15]	2003	22	100% (9/9)	92% (12/13)	90% (9/10)	100% (12/12)
Kwon et al. [16]	2002	52	100% (39/39)	100% (13/13)	100% (39/39)	100% (13/13)
Lavonius et al. [17]	2001	24	100% (11/11)	69% (9/13)	73% (11/15)	100% (9/9)
Taylor et al. [18]	2001	49	100% (26/26)	91% (21/23)	93% (26/28)	100% (21/21)
Schachter et al. [19]	2000	67	100% (33/33)	88% (30/34)	89% (33/37)	100% (30/30)
Velasco et al. [20]	2000	33	100% (22/22)	82% (9/11)	92% (22/24)	100% (9/9)
Norton et al. [21]	1999	50	100% (26/26)	92% (22/24)	93% (26/28)	100% (22/22)
Minnard et al. [22]	1998	90	100% (40/40)	98% (49/50)	98% (40/41)	100% (49/49)
Champault et al. [23]	1997	26	100% (5/5)	100% (21/21)	100% (5/5)	100% (21/21)
Pietrabissa et al. [24]	1996	21	100% (13/13)	100% (8/8)	100% (13/13)	100% (8/8)
Bemelman et al. [6]	1995	70	100% (22/22)	33% (16/48)	41% (22/54)	100% (16/16)
John et al. [4]	1995	38	92% (11/12)	88% (23/26)	79% (11/14)	96% (23/24)
Murugiah et al. [25]	1993	12	100% (5/5)	86% (6/7)	83% (5/6)	100% (6/6)

## Abbreviations

CT: Computed tomography
DL: Diagnostic laparoscopy
DLUS: Diagnostic laparoscopy with ultrasound
LUS: Laparoscopic ultrasound
MDCT: Multidimensional computed tomography
SI: Standard imaging.
